# Haemoadsorption in infective endocarditis: a systematic review

**DOI:** 10.1007/s12055-024-01701-0

**Published:** 2024-02-20

**Authors:** Matthias Thielmann, Daniel-Sebastian Dohle, Martin Czerny, Nikolaos Bonaros, Daniel Wendt, Thierry Folliguet, Christophe Baufreton, Guillaume Lebreton

**Affiliations:** 1 Department of Thoracic and Cardiovascular Surgery, West German Heart & Vascular Center Essen, Hufelandstr. 55, 45122 Essen, Germany; 2 University Medical Center of the Johannes Gutenberg University Mainz, Mainz, Germany; 3 Department of Cardiovascular Surgery, Medical Center University of Freiburg, Faculty of Medicine, University of Freiburg, Freiburg, Germany; 4 Department of Cardiac Surgery, Medical University of Innsbruck, Innsbruck, Austria; 5 CytoSorbents Europe GmbH, Berlin, Germany; 6 Department of Cardiac Surgery, Henri Mondor Hospital, Paris, France; 7 Department of Cardiovascular and Thoracic Surgery, University Hospital, Angers, France; 8 Department of Thoracic and Cardiovascular Surgery, Pitié-Salpêtrière University Hospital, Paris, France

**Keywords:** Haemoadsorption, Infective endocarditis, CytoSorb®, Cardiac surgery

## Abstract

Infective endocarditis surgical patients suffer from high rates of severe complications such as systemic inflammatory response, septic shock, and multi-organ failure leading to high mortality. Systemic inflammatory response based on cytokines as messengers plays an important role in these patients. The concept of intraoperative haemoadsorption has been proposed to remove such elevated cytokines in patients undergoing cardiac surgery for infective endocarditis. Haemoadsorption offers the possibility to stabilise haemodynamics, reduce sepsis-related mortality, and protect organ function. However, until now, there has been no general opinion and consensus regarding the clinical effectiveness of adjunctive intraoperative haemoadsorption in infective endocarditis. Therefore, we reviewed the current literature evaluating haemoadsorption in infective endocarditis patients undergoing cardiac surgery. The review was registered at PROSPERO (CRD42023457632).

**Supplementary Information:**

The online version contains supplementary material available at 10.1007/s12055-024-01701-0.

## Introduction

Complicated acute infective endocarditis (IE) is a life-threatening disease that requires open heart surgery with the use cardiopulmonary bypass (CPB), which may be complicated by vasoplegia, and, in worst-case scenarios, vasoplegic shock due to systemic inflammatory response syndrome (SIRS) [1, 2]. A systemic inflammatory response in combination with systemic infection caused by different pathogens is in general orchestrated by cytokines as messengers resulting in a cytokine release syndrome or the so-called cytokine storm [3]. Sepsis and subsequent organ failure are an important cause of death especially in high-risk IE patients undergoing cardiac surgery for IE [4, 5].

Intraoperative haemoadsorption has been proposed to remove inflammatory mediators and might be supportive in IE patients operated on with CPB [6]. Until now, there is no general opinion and consensus about the clinical effectiveness of adjunctive intraoperative haemoadsorption in IE.

The aim of the present systematic review is to summarise the current published knowledge of intraoperative haemoadsorption in the setting of IE.

## Methods

### Literature search

A systematic search of the PubMed database was performed on 28 November 2023, using the following key search words: “endocarditis” AND “hemoadsorption” OR “hemoadsorbtion” OR “hemadsorption” OR “hemadsorbtion” OR “haemoadsorption” OR “haemoadsorbtion” OR “haemadsorption” OR “haemadsorbtion”. Moreover, the CytoSorbents literature database was also evaluated (https://literature.cytosorb-therapy.com/). Eligibility of studies for inclusion was cross-checked by senior authors (M.T. and C.B.). Full texts of the remaining articles were then screened. During the literature screening and selection process, the principles derived from the Preferred Reporting Items for Systematic Reviews and Meta-Analyses (PRISMA) guidelines with an intention of preserving an objective approach were followed (Supplement). The systematic review was registered at PROSPERO (CRD42023457632).

### Inclusion and exclusion criteria

Only studies evaluating the intraoperative use of haemoadsorption (CytoSorb®,CytoSorbents, Princeton, NJ, USA) in cardiac surgery for IE were included. Manuscripts were considered for inclusion if they were in the English language and published in the last 10 years. Single case reports, letters to the editor, and protocol publications were excluded.

## Results

### Summary of included papers

**Fig. 1:**
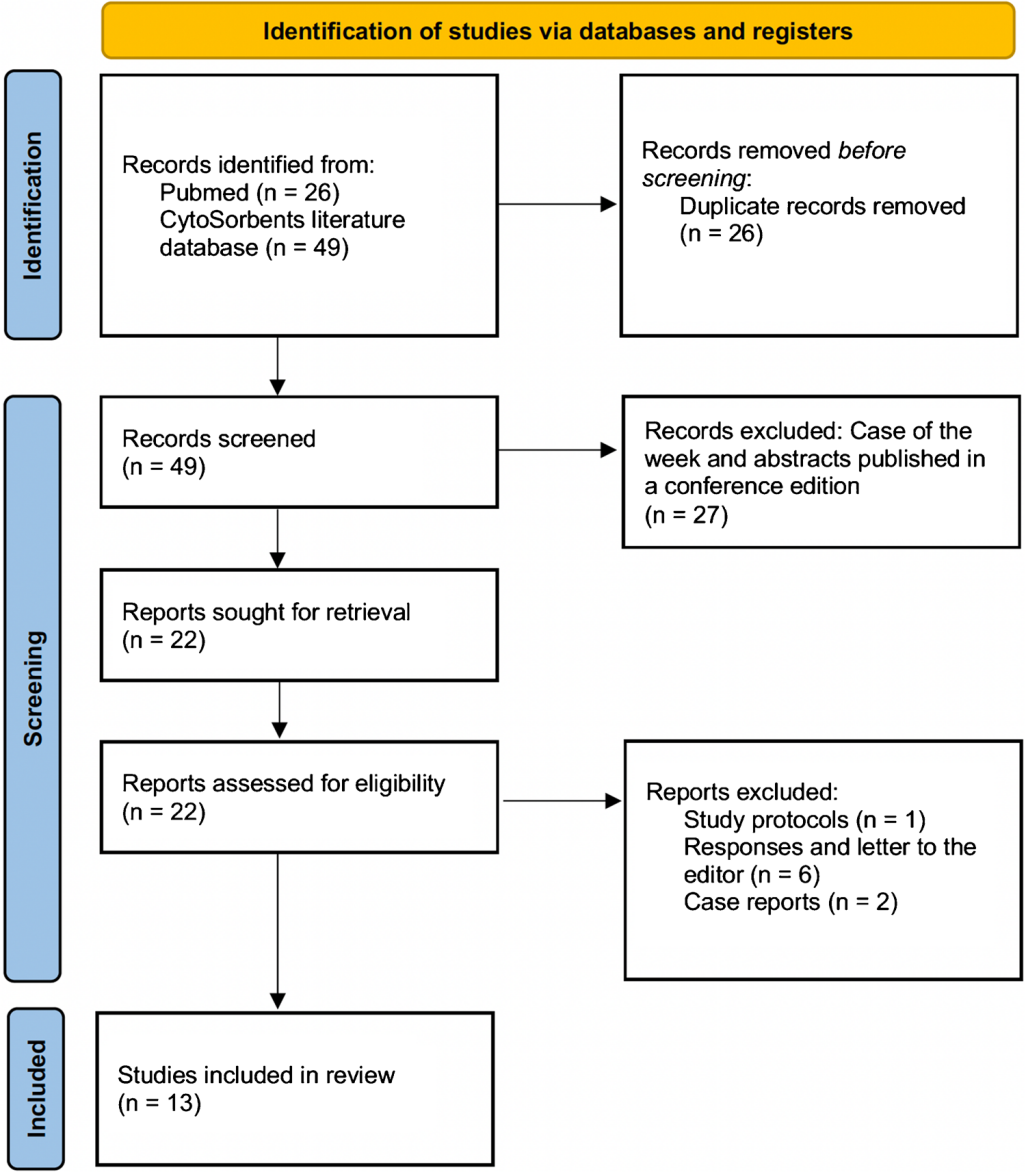
Identification of studies via databases and registers

A total of 13 studies could be extracted evaluating IE and haemoadsorption in the cardio-surgical field including three randomised controlled trials (RCTs) (Fig. 1). All studies were published between 2017 and 2023. Of these, a total of 6 studies were observational trials, with three of them including dedicated propensity score matching. One study presented basic research data on IE and another study presented a theoretical budget impact analysis. One study was a meta-analysis of the use of haemoadsorption in the field of cardiac surgery also including IE.

In chronological order, all 13 studies are summarised: The first experience with intraoperative haemoadsorption in IE was published by Träger et al. in 2017 [6]. The authors described a case series of 39 patients presenting with proven IE being operated on in combination with intraoperative CytoSorb® haemoadsorption. The authors showed a marked reduction in inflammatory parameters (interleukin—IL-6 and IL-8) and quick haemodynamic stabilisation with a rapid decrease in the need for postoperative vasopressors. Träger et al. compared their results to 28 historical control patients and the authors could show a shorter intensive care unit (ICU) stay in the intervention group (median 5.0 days vs. 7.5 days). In 2019, Kuhne et al. evaluated the postoperative continuation of CytoSorb® treatment in IE patients [7]. In 10 IE patients, only intraoperative haemoadsorption was performed, whereas in another 10 IE patients, the treatment was continued postoperatively in the ICU using continuous renal replacement therapy (CRRT). The authors showed that although the intraoperative plus postoperatively treated patients had higher risk scores and a more pronounced disease severity, they had similar results compared to the only intraoperative haemoadsorption group. It should be acknowledged that Kuhne et al. did not use new fresh adsorbers to continue postoperatively. The authors flushed the intraoperatively used and saturated adsorbers and implemented these adsorbers into the CRRT, which is usually not recommended by the manufacturer.

A retrospective case-controlled analysis evaluated the intraoperative use of haemoadsorption in native mitral valve IE patients, which was published in 2020 by Haidari et al. [8]. The authors compared 30 haemoadsorption patients to 28 controls without intraoperative haemoadsorption. Interestingly, the authors could show a statistically significant reduction in the levels of postoperative inotropic support in-line with improved systemic vascular resistance. They could also prove for the first time both a significant reduction in postoperative sepsis and in sepsis-associated mortality according to the SEPSIS-3 guidelines [9]. However, these findings did not translate into a statistically significant difference in 30-day mortality.

A retrospective case-controlled study by Santer et al. evaluated the use of intraoperative haemoadsorption by inversed probability treatment weighting [10]. A total of 41 patients were treated by intraoperative haemoadsorption compared to 200 historical controls (time interval: 2009 to 2019). Since various intensive care strategies changed over this 10-year period, results were also adjusted for the impact of time. Santer et al. [10] observed different results compared to Haidari et al. [8]: The authors presented higher inotropic support in the postoperative period (higher noradrenaline and milrinone demand) and interestingly more red blood cell and platelet transfusions. Moreover, higher bleeding events with a higher incidence of reoperations resulting in prolonged hospitalisation were described. Regarding their primary outcome parameter of in-hospital mortality, no difference could be observed.

The first RCT evaluating the application of haemoadsorption in IE included in total 20 patients [11]. The primary endpoint parameter was the postoperative course of cytokine levels as well as inflammatory parameters. The treatment group received postoperative continuation of the haemoadsorption therapy for 24 h. The authors changed the adsorbers every 8 h based on an estimated saturation of the device resulting in one intra- and three postoperative adsorbers (overall 4 adsorbers per patient). The overall risk as calculated by the European System for Cardiac Operative Risk Evaluation (EuroSCORE)-II in their cohort was 8.5% (median) in the haemoadsorption group compared to 3.6% in the controls (*p* = 0.39). Asch et al. concluded that haemoadsorption therapy did not result in a reduction of inflammatory parameters nor result in an improvement in haemodynamic parameters in patients operated on for IE.

In 2022, a small single RCT was published by a group from Gothenburg, Sweden [12]. A total of 19 patients with IE requiring cardiac surgery were randomised to receive either intraoperative CytoSorb® whilst on the CPB circuit (10 pts) or standard care (9 pts). The authors showed that the accumulated dose of noradrenaline was at least doubled in the control group postoperatively at all time points after surgery; however, this was statistically non-significant due to the low patient numbers included. At 3 h postoperatively, the median accumulated amount of noradrenaline was 16 µg vs. 36 µg; at 6 h, 28 µg vs. 82 µg; and after 12 h, 32 µg vs. 112 µg. After 48 h, it was 36 µg vs. 261 µg (*p* = 0.09), which corresponds to an almost sevenfold increased noradrenaline amount in the controls. Duration of noradrenaline dose was also numerically longer in the control group (median 6 h vs. 48 h). In contrast to Santer et al. [10], Holmen et al. could show in their small RCT a significantly lower need for red blood cell transfusions in the CytoSorb® group (285 vs. 1940 mL, *p* = 0.03). The amount of transfused plasma and platelets was also greater in the control group. There was also a non-significant trend towards a shorter time on the ventilator in the CytoSorb® group [12].

Just a few weeks after Holmen et al. published their small RCT, the Revealing Mechanisms and Investigating Efficacy of Hemoadsorption for Prevention of Vasodilatory Shock in Cardiac Surgery Patients With Infective Endocarditis (REMOVE) trial was published [13]. In this multi-centre, non-blinded control trial, 288 IE patients were randomly assigned to either intraoperative haemoadsorption use with CytoSorb® (142 pts) or standard treatment (146 pts). The REMOVE trial failed to show a reduction in the Sequential Organ Failure Assessment (SOFA) score (being the primary endpoint parameter) in the postoperative course in the haemoadsorption group. However, the REMOVE trial could nicely show that all important cytokines, including cell-free deoxyribonucleic acid (DNA) and midregional pro-adrenomedullin (MR-proADM), were highly significantly reduced in the haemoadsorption group (first 50 randomised patients being evaluated). Results showed that there was no difference in the mortality or duration of mechanical ventilation, use of vasopressors, or renal replacement therapy.

In the same year, another study by Haidari et al. evaluated the clinical effects of intraoperative haemoadsorption in high-risk patients [14]. High-risk patients with IE and intraoperative haemoadsorption were compared to patients without haemoadsorption and propensity score matching was applied. After matching, 70 high-risk patients were included (35 in each arm). The endpoints were the incidence of postoperative sepsis, sepsis-associated mortality, and in-hospital mortality. Additionally, postoperative vasopressor need, systemic vascular resistance index, and SOFA scores were compared. Rates of postoperative sepsis were similar (14 patients in the haemoadsorption group and in 16 patients in the control group, *p* = 0.629). Four patients died due to postoperative sepsis in the haemoadsorption group, while 11 postoperative septic patients died in the control group, *p* = 0.041. In-hospital mortality was 34% in the haemoadsorption group versus 43% in the control group, *p* = 0.461. On ICU admission and the first postoperative day, the cumulative vasopressor need (noradrenaline and adrenaline) was 0.17 versus 0.25 µg/kgBW/min, *p* = 0.123 and 0.06 vs. 0.11 µg/kgBW/min, *p* = 0.037. Interestingly, this study also evaluated the systemic vascular resistance index in a comprehensive manner (as measured by pulmonary Swan-Ganz catheter). The vascular resistance was significantly improved in the haemoadsorption group on ICU admission 1448 versus 941 dyn*s*cm-5, *p* = 0.013. The postoperative course of SOFA score normalised significantly faster (*p* = 0.01) in the haemoadsorption group. Moreover, respiratory failure requiring reintubation occurred in 20 pts (6 in the haemoadsorption group and 14 in the control group, *p* = 0.034).

A few months later, another propensity score-matched analysis was published by Kalisnik et al. [15]. The objective was to assess the efficacy of intraoperative haemoadsorption in active left-sided native (135 pts) and prosthetic IE (67 pts). Active IE was defined as an ongoing infection under antibiotic therapy. Patients with intraoperative haemoadsorption were compared to patients without haemoadsorption (controls) over a 6-year period. Ninety-nine patients received intraoperative haemoadsorption inserted into the CPB circuit and 103 patients did not. Ninety-nine propensity-matched pairs were selected for final analyses, resulting in an overall cohort of 198 patients. The authors could show that postoperative sepsis and sepsis-related mortality were reduced in the haemoadsorption group (22.2% vs. 39.4%, *p* = 0.014 and 8.1% vs. 22.2%, *p* = 0.01, respectively). In-hospital mortality tended to be lower in the haemoadsorption group (14.1% vs. 26.3%, *p* = 0.052). Key predictors for sepsis-associated mortality and in-hospital mortality were preoperative inotropic support, lactate levels 24 h after surgery, C-reactive protein levels on postoperative day 1, chest tube output, cumulative inotropes and white blood cell counts on postoperative day 2, and new onset of dialysis. Of note, multivariate regression analysis revealed, for the first time, that intraoperative haemoadsorption with CytoSorb® as a preventative measure was significantly associated with lower sepsis-associated, as well as in-hospital mortality.

More recently, a dual-centre trial evaluated the impact of intraoperative haemoadsorption in patients with IE based on *Staphylococcus aureus* [16]. The authors compared 55 controls to 75 patients with intraoperative haemoadsorption undergoing cardiac surgery. The author’s hypothesis was that using CytoSorb® in this setting was for the removal *of Staphylococcus aureus* endotoxin by the adsorber, which might be the cause of postoperative vasoplegia and poor outcomes. There were no differences in the demographics. The mean EuroSCORE-II for both groups was 11.9% and 12.0%, indicating a high-risk surgical population. Results showed improved postoperative haemodynamic stabilisation with a significantly decreased vasoactive inotropic score (VIS) in the haemoadsorption group at all time points from 6 to 72 h postoperatively. Importantly, sepsis-related mortality (8.0% vs. 22.8%, *p* = 0.02) and 30-day (17.3% vs. 32.7%, *p* = 0.03) and 90-day overall mortality (21.3% vs. 40%, *p* = 0.03) were also significantly lower in the haemoadsorption group. New, postoperative renal failure requiring haemodialysis developed in 38 patients, 16 in the haemoadsorption group and 22 in the control group (*p* = 0.03).

### Basic research

A basic research analysis by Piskovatska et al. evaluated the adsorbed proteins during haemoadsorption [17]. Interestingly, the authors stored the intraoperatively used adsorbers from the REMOVE trial [13]. Directly after disassembling, the polymer beads were extracted from the adsorber and were stored in 5.0-mL Eppendorf tubes at − 20 °C until protein extraction. Proteins were thereafter eluted from the polymer beads and a pool of 4 different elutions from 4 different adsorbers was used to test the effect on the endothelium. Most importantly, the authors could show that the elution had a severe effect on the viability of human aortic endothelial cells. Moreover, the same results were observed when applying the same elution to a standardised wound healing panel. In comparison to the before-mentioned results when the elution was used, no cell viability alterations were observed when commercially available serum was added to the test setup. Along with these results, the authors showed a broad spectrum of protein removal by haemoadsorption. Therefore, the authors stated that intraoperative haemoadsorption is capable of binding diverse detrimental proteins. Moreover, the authors concluded that “*Material eluted from the* CytoSorb® *matrix used for hemoadsorption in patients with infective endocarditis causes dose-dependent and significant reduction in viability and migratory capacity of cultured endothelial cells*.”

### Budget impact analysis

Recently, a theoretical budget impact model was published by Rao et al. [18]. This theoretical model was based on the data from Träger et al. showing a difference in postoperative ICU stay in a group of IE patients with intraoperative haemoadsorption [6]. This theoretical model calculated all costs for the therapy of intraoperative haemoadsorption based on a potential annual population of 550 patients for the whole system in Germany. The model resulted in potential cost savings of 2298€ in the base-case scenario without specific reimbursement of the therapy, which increased up to 3804€ per patient in the case of full device-specific reimbursement. In conclusion, the authors stated that, despite being a theoretical model, intraoperative haemoadsorption might lead to important cost savings.

### Review and meta-analysis

By using the above-mentioned keywords for the systematic search, a systematic review and meta-analysis by Naruka et al. was retrieved [19]. The authors did not restrict the types of cardiac surgery, including cardiac transplantation and IE. They conducted their analysis on the main outcomes (operative mortality, ventilation duration, ICU, and hospital stays) and day 1 inflammatory marker levels postoperatively. A total of 15 studies were included for the final analysis (eight RCTs and 7 observational studies) showing no evidence of publication bias. Although all forms of adsorption therapies were allowed to be included in the analysis, 12 of the 15 studies were using CytoSorb®. Subgroup analysis of non-elective cardiac surgery across all studies (emergency and IE) significantly favoured haemoadsorption in terms of 30-day mortality (*p* = 0.01) and shorter ICU stay (*p* = 0.001), while comparing haemoadsorption and controls across all studies, including elective surgeries, showed no significant difference in this regard. According to the authors, this illustrates the fact that cytokine adsorption may be preferentially more effective in patients with high inflammatory response, such as with IE or cardiac transplant patients or high-risk and longer-lasting surgeries. The authors finally conclude that a significant reduction in 30-day mortality and ICU stay could be obtained by using haemoadsorption therapy during non-elective cardiac surgery, especially emergency surgery and in patients with higher inflammatory burdens such as in IE.

**Fig. 2:**
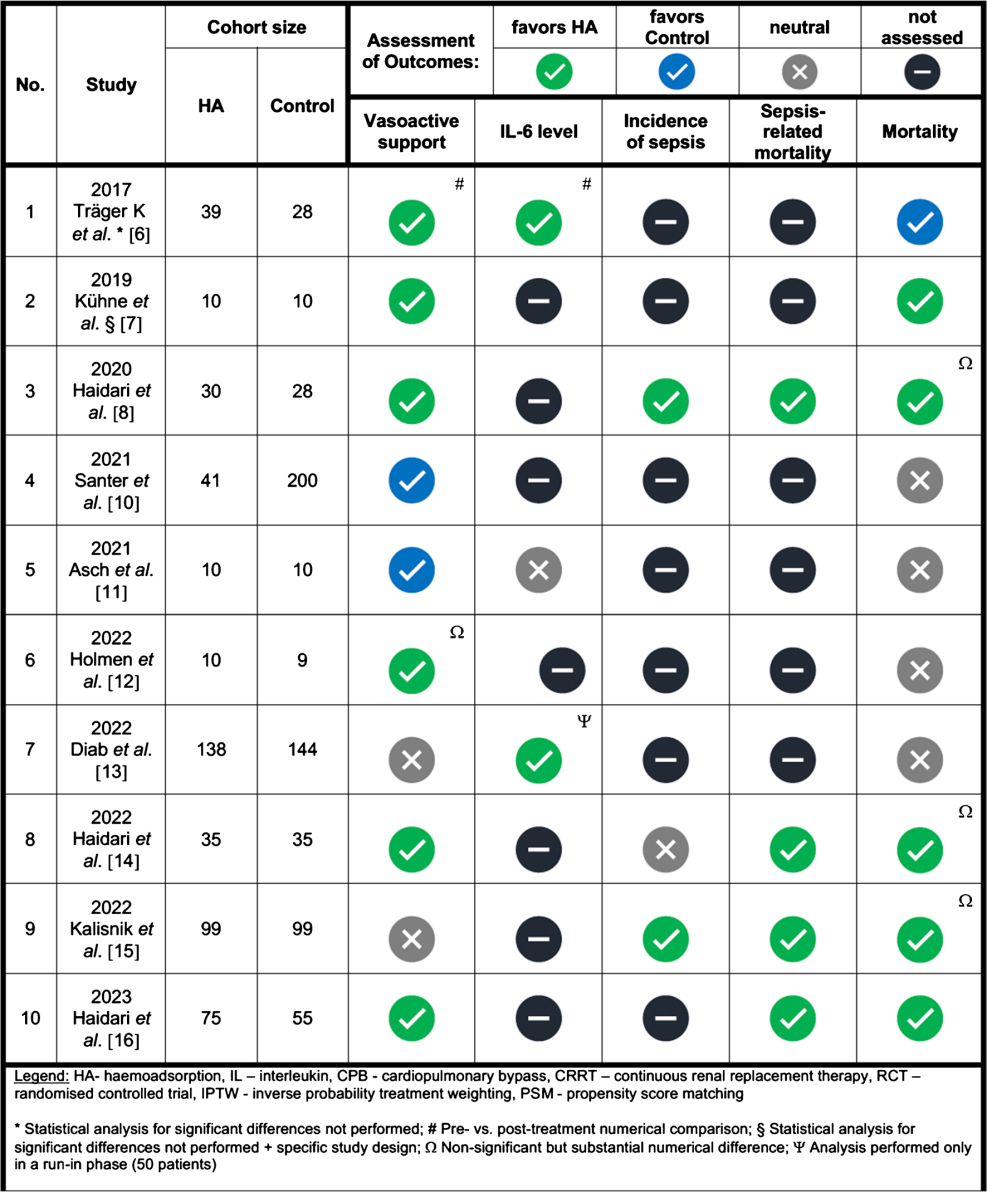
A summary of the relevant and evaluated outcome parameters

Figure 2 summarises all relevant and evaluated outcome parameters.

## Discussion

Herein, we critically assessed and summarised the currently available literature on the use of haemoadsorption in patients undergoing cardiac surgery for IE. Notably, the current narrative review evaluated a total of 13 studies and CytoSorb® was the most used haemoadsorption device in the field of IE. Overall, the currently available evidence regarding the use of haemoadsorption in IE is mixed, but in aggregate suggests limited value with use in routine elective surgery and low-risk patients.

However, and importantly, the mode of action and final proof of concept of cytokine removal was shown in an RCT by Jansen et al. [20]. The authors could show a highly significant removal of all cytokines in a cohort of healthy volunteers treated by lipopolysaccharides resulting in a “*cytokine storm-like*” situation with IL-6 levels reaching up to 3000 pg/mL. Since some articles included into the current review could not detect a significant removal of cytokines, the study by Jansen et al. provides the irrevocable validation of an effective attenuation and removal of circulating cytokines by haemoadsorption [20].

The innovation and application of haemoadsorption in IE has developed over time starting from a first case series to several observational studies to a wider application and finally evaluation in three RCTs. In some studies, a reduced rate of sepsis and/or sepsis-associated mortality has been shown, whereas the REMOVE trial showed rather neutral results in this regard [13]. Others could prove significant reduction of plasma cytokine levels (which has been also proven by the REMOVE trial); however, the REMOVE authors stated that routine use of CytoSorb® during routine cardiac surgery for IE was not justified. A mortality benefit has been shown only by a most recent analysis by Haidari et al. in high-risk IE patients with a proven *Staphylococcus aureus* infection [16]. A potential explanation for this benefit on mortality could be that the *Staphylococcus aureus* toxic shock toxin and haemolysin are removed by intraoperative haemoadsorption. This was published by Gruda et al. in a benchtop analysis, showing significant removal of the two proteins, which are playing a crucial role in the development of vasoplegia [21]. On a separate note, Piskovatska could show the harmful effects of an elution derived from the stored REMOVE adsorbers on cell viability [17]. A growing body of studies have shown a reduction in inotropic support in the postoperative period in IE patients being treated intraoperatively. This was also proven by a small RCT from Sweden where the authors stated that “*Although the primary endpoint for the study (amount of noradrenaline used 24 and 48 h postoperatively) did not reach statistical significance, the results show a trend towards beneficial outcomes with the use of* CytoSorb®*, with better hemodynamic stability in the intensive care unit, and lesser amounts of transfused blood product postoperatively*” [12]. In contrast to this, others have not shown a reduced vasopressor support postoperatively in IE patients in whom haemoadsorption was used intraoperatively [10]. Moreover, there is still an ongoing debate with conflicting evidence [7, 11] as to if haemoadsorption should be continued postoperatively after endocarditis surgery. Boss et al. showed in a high-risk group of patients undergoing cardiac surgery (including IE) the benefit of the postoperative use of haemoadsorption by reduction of the postoperative vasopressor need in line with better outcomes compared to the SOFA- and Acute Physiology and Chronic Health Evaluation (APACHE) score-predicted mortality [22].

The REMOVE trial is by far the largest RCT evaluating the use of intraoperative haemoadsorption in IE patients thus far [13]. The primary outcome of the REMOVE trial did not show any statistically significant difference in the preoperative versus postoperative SOFA scores. However, interestingly, the REMOVE trial showed on the one hand, that IL-6 levels increased significantly depending on the length of the pump-run, and on the other hand, that intraoperative haemoadsorption could significantly reduce circulating cytokines. However, within the REMOVE trial, the cytokine reduction did not translate into better survival. Unfortunately, the REMOVE trial did not present specific details on the postoperatively inotropic support of both treatment arms. Only the cardiovascular sub-score of the SOFA score was presented, which showed no difference. The results of the REMOVE trial could potentially be explained by the fact that only about 10% of the REMOVE patients with about 30% elective cases were not in a critical state, and only 1.4% of the treatment group showed a preoperative disruptive shock. Others reported a critical preoperative status ranging between 10.0 and 16.7% [8]. This goes in line with the preoperative inflammation status of both groups in the REMOVE trial. Of note, the preoperative IL-6 levels within the REMOVE trial in both arms did not exceed 50 pg/mL preoperatively. One could speculate that these patients were not presenting with a “*hot*” endocarditis.

In the current review, three RCTs have been included showing mixed outcomes. In the medical community, usually RCTs are seen as the highest quality studies aiming at collecting scientific evidence at the highest level. Regarding haemoadsorption in IE, a reduction of the inflammatory response by a significant reduction of IL-6 levels has been proven by some RCTs. However, in regard to other outcomes, it has to be discussed if RCTs are sometimes inappropriate in terms of sample size or the chosen primary endpoints. For instance, is mortality or the SOFA score an appropriate primary endpoint for a trial on such a complex topic? This has been also witnessed in other specialties, such as intensive care medicine, where many RCTs have not demonstrated any beneficial effects of an intervention. The complex entity IE in combination with haemoadsorption also has to deal with timing, proper endpoint selection, and heterogeneous populations. Taking this into account, it makes it very difficult to conduct RCTs in this realm.

Another important topic which should be discussed is the unintended removal of other substances, such as antibiotics, which is the case for all extracorporeal circuits. This side-effect is of utmost importance especially in the field of IE. In a group of septic shock patients, Scharf et al. showed a clinically significant adsorption of vancomycin with CytoSorb® use [23]. Therefore, the authors recommended the administration of an additional dose of 500 mg vancomycin over 2 h to avoid subtherapeutic vancomycin exposure and stringent therapeutic drug monitoring (TDM). TDM has also been recommended by others [24].

In summary, since the results of intraoperative haemoadsorption in patients undergoing cardiac surgery for IE are mixed, appropriate selection criteria should be developed and used to target the right patients. First, the therapy goal should be identified, which could potentially be a reduction in postoperative vasopressor support. A second option would be to initiate haemoadsorption therapy in patients with IE based on an individualised approach based on cytokine or inflammatory plasma levels. Finally, also the timing and dosing of the therapy should be further evaluated since there are currently no recommendations for which IE patients the therapy should be prolonged in the postoperative period.

Since the therapeutic goal of haemodynamic stability seems to be a reasonable endpoint; e.g., the vasoactive inotropic score should be routinely calculated. Moreover, to capture real-world data on the current international strategies and regimes in the treatment of IE, the SURgical Registry of Infective ENDocarditis in EuRope (SURRENDER) was designed (NCT05563662).

In conclusion, from a mechanistic point of view, the concept of cytokine removal by haemoadsorption in IE patients has been proven [13, 17]. Studies have also suggested haemodynamic stabilisation in the postoperative period for IE patients in whom haemoadsorption was applied intraoperatively. In summary, much more data is needed to better define appropriate selection criteria (e.g., patients with an ongoing infection under antibiotic therapy) and more information regarding the timing and dosing of haemoadsorption therapy in the field of IE.

### Supplementary Information

ESM 1 (PDF 822 KB)

## Data Availability

N/A (review).

## Code availability

The current work was registered at PROSPERO (CRD42023457632).
